# Isolation and Identification of *Andrographis paniculata* (*Chuanxinlian*) and Its Biologically Active Constituents Inhibited Enterovirus 71-Induced Cell Apoptosis

**DOI:** 10.3389/fphar.2021.762285

**Published:** 2021-12-08

**Authors:** Wen-Wan Chao, Yueh-Hsiung Kuo, Bi-Fong Lin

**Affiliations:** ^1^ Department of Nutrition and Health Sciences, Kainan University, Taoyuan, Taiwan; ^2^ Department of Chinese Pharmaceutical Sciences and Chinese Medicine Resources, China Medical University, Taichung, Taiwan; ^3^ Department of Chemistry, National Taiwan University, Taipei, Taiwan; ^4^ Department of Biochemical Science and Technology, National Taiwan University, Taipei, Taiwan

**Keywords:** *Andrographis paniculata*, antiviral activity, cytopathic effect, enterovirus 71, rhabdomyosarcoma cells

## Abstract

**Aim:** *Andrographis paniculata* (Burm. f.) Nees (also known as *Chuanxinlian* in Chinese) of Acanthaceae family is one of the Chinese herbs reputed to be effective in the treatment of inflammation, infection, cold, and fever. Enterovirus 71 (EV71) is one of the most important enteroviruses that cause hand, foot, and mouth disease (HFMD) accompanied with neurological complication.

**Methods:** To explore an anti-infective Chinese herb medicine, pure compounds isolated or synthesized analogues from *A. paniculata* (AP) ethyl acetate (EtOAc) extract are used to explore their anti-EV71-induced cytotoxicity. The antiviral activity was determined by cytopathic effect (CPE) reduction, and sub-G1 assays were used for measuring lysis and apoptosis of EV71-infected rhabdomyosarcoma (RD) cells. IFNγ-driven luciferase reporter assay was used to evaluate their potential roles in activation of immune responses.

**Results:** Our data showed that EV71-induced sub-G1 phase of RD cells was dose dependently increased. Highly apoptotic EV71-infected RD cells were reduced by AP extract treatment. Ergosterol peroxide (**4**) has the most anti-apoptotic effect among these seven compounds. In addition, 3,19-*O*-acetyl-14-deoxy-11,12-didehydroandrographolide (**8**) synthesized from acetylation of compound **7** showed significantly better antiviral activity and the lowest sub-G1 phase of 6%–18%. Further investigation of IFNγ-inducer activity of these compounds showed that compounds **3**, **6**, **10**, **11**, and **12** had significantly higher IFNγ luciferase activities, suggesting their potential to promote IFNγ expression and thus activate immune responses for antivirus function.

**Conclusion:** Our study demonstrated that bioactive compounds of AP and its derivatives either protecting EV71-infected RD cells from sub-G1 arrest or possessing IFNγ-inducer activity might be feasible for the development of anti-EV71 agents.

## 1 Introduction

*Andrographis paniculata* (Burm. f.) Nees (also known as *Chuanxinlian* in Chinese) of Acanthaceae family, native to Taiwan, Mainland China, and India, is a medicinal plant widely used for anti-inflammatory, antipyretic, antiviral, and detoxifying purposes. The leaves and aerial parts of *A. paniculata* (AP) have been used in traditional Chinese medicine (TCM). It has been considered as a “cold” herb and used to get rid of body heat and toxins. Earlier works have identified many important ingredients in the plant, including diterpenes, flavonoid, and stigmasterols (Chao et al., 2010; Chao and Lin, 2010; Saxena et al., 2010; Kumar et al., 2021). It is commonly employed for “clearing heat and resolving toxicity.” Typical symptoms of “heat and toxicity” include swollen and painful gums, associated with inflammation, cancer, and virus-related diseases (Wang et al., 2016; Jiang et al., 2021).

There have been several reports on the effects of these ingredients on antivirus activity. Andrographolide, neoandrographolide, and 14-deoxy-11,12-didehydroandrographolide (**7**) isolated from AP demonstrated virucidal activity against herpes simplex virus 1 without significant cytotoxicity (Wiart et al., 2005). Andrographolide was also shown to inhibit the expression of Epstein–Barr virus lytic proteins during the viral lytic cycle in infected P3HR1 cells, *via* inhibiting the production of mature viral particles without harm to the cells (Lin et al., 2008). Anti-influenza activity of andrographolide and its derivatives was also demonstrated in mice infected with H1N1, H9N2, or H5N1, as well as in infected canine kidney cell line Madin–Darby canine kidney (MDCK) cells (Chen et al., 2009). They further synthesized 14-α-lipoyl andrographolide derived from andrographolide and found it more effective against avian influenza A virus H9N2 and H5N1 and human influenza A H1N1 virus *in vitro*. Jiang et al. (2009) also showed that synthesized andrographolide analogue, 14-glycinyl andrographolide hydrochloride, exerted more potent antibacterial activity. These studies suggest that chemical modification of bioactive compounds isolated from plants is worthy of study to improve the efficacy of anti-infection (Jiang et al., 2009).

Enterovirus type 71 (EV71) infection is one of the serious public health problems, especially in Asia. The pathogen was originally recognized in l969 in California with subsequent outbreaks in other parts of the United States. Since then, outbreaks have been noted in Australia, Japan, Korea, Malaysia, Singapore, Vietnam, and China (Ho, 2000; Chung et al., 2010). EV71 is a single positive-stranded RNA virus that belongs to the *Enterovirus* genus of the *Picornaviridae* family. EV71 infection might be asymptomatic or might cause diarrhea, rashes, vesicular lesions on the hands and feet, and oral mucosa (hand, foot, and, mouth disease (HFMD)), which are typically found in infants and children. Sometimes, infection can lead to severe herpangina, aseptic meningitis, encephalitis, or myocarditis, which might be fatal in infants and children. The viruses are spread through contact with virus-containing body fluids, respiratory droplets, and feces. There is currently no vaccine or specific medication for EV71 infections, though antivirus drug ribavirin for hepatitis was reported to reduce mortality of EV71-infected mice (Li et al., 2008).

HFMD is caused mainly by an accumulation of damp heat and toxicity in the body, and therefore its treatment may involve the usage of heat-clearing and detoxifying medicines (Wang et al., 2016). Therefore, in this study, we investigated the anti-EV71-induced cytotoxicity of 12 compounds isolated and modified from AP (Chao et al., 2010). EV71 infection induces cytopathic effect (CPE) on the host cells, such as neuroblastoma, colorectal adenocarcinoma, and rhabdomyosarcoma (RD) cells, leading to eventual cell death (Bai et al., 2019). Therefore, the antiviral activity of the fractions and compounds from AP ethyl acetate (EtOAc) extract against EV71 was examined by CPE in EV71-infected RD cells.

In addition, interferon (IFN)-mediated antiviral responses are very important to host defense against viral infection. Both type I IFNs (IFNα/β*)* and type II IFN (IFNγ) play an important role in controlling EV71 infection and replication. Administration of IFN inducer was reported to protect the mice against EV71 infections *via* higher IFNα production (Liu et al., 2005; Lin et al., 2019). In severe EV-A71 infection, the increase of IFNγ inducible protein-10 subsequently elevating expressions of IFNγ is crucial for virus clearance and survival of EV71-infected mice (Shen et al., 2013). Therefore, we also investigated the anti-EV71-induced cytotoxicity of these 12 compounds from AP (Chao and Lin, 2010) by evaluating their IFN-inducing activity using an IFNγ-luciferase reporter assay (Chao et al., 2009). Therefore, screening for IFN inducers or immune-stimulatory compounds from medicinal plant is a practical approach to identify potent antiviral agents.

## 2 Article Types

Original Research Article.

## 3 Materials and Methods

### 3.1 Plant Material

**FIGURE 1:**
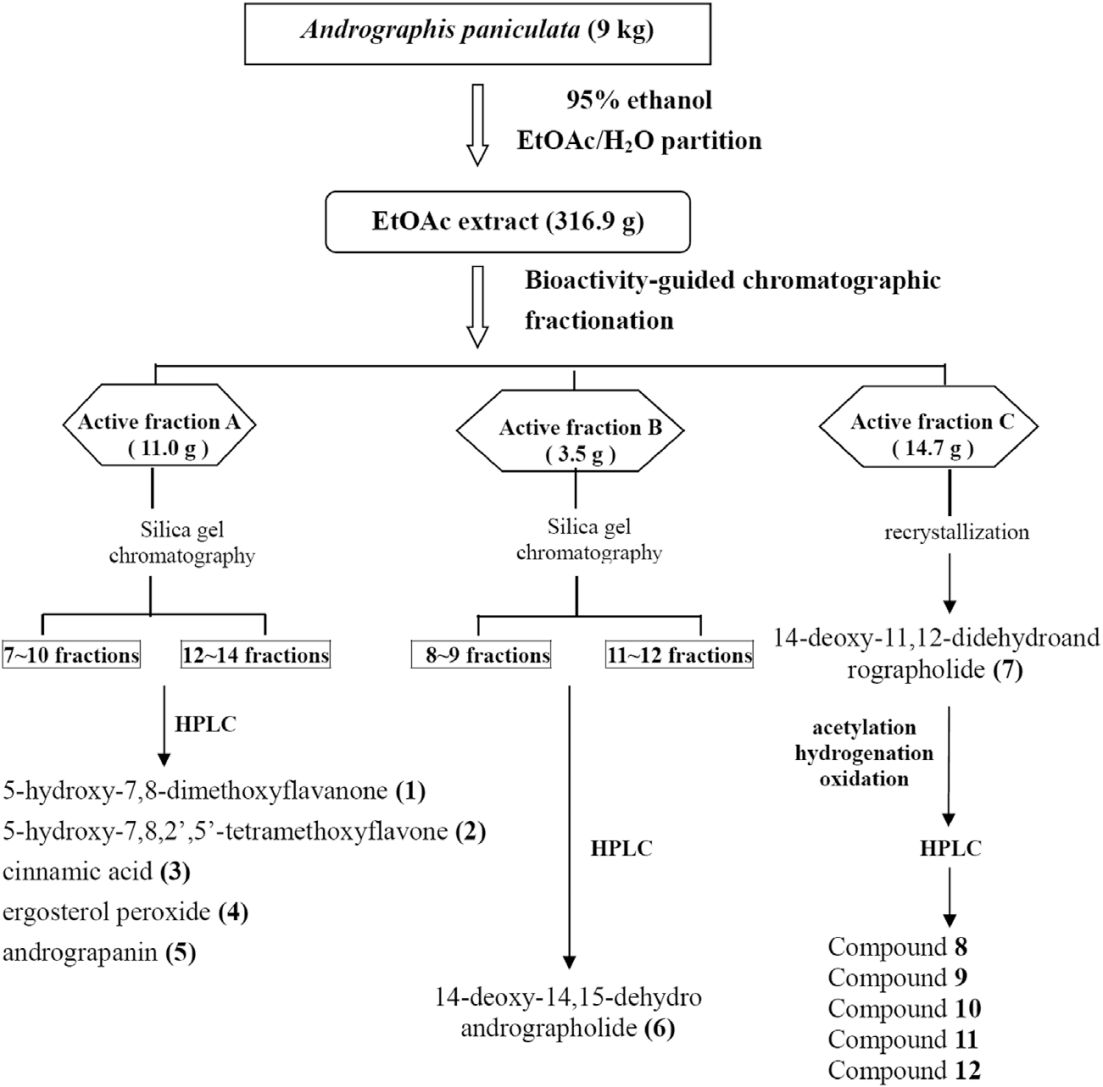
The extraction procedure for the separation and identification of *Andrographis paniculata* EtOAc extract. Compounds tested for anti-EV71-induced cytotoxicity are enumerated with Arabic numbers (bold). EtOAc, ethyl acetate; EV71, enterovirus 71.

*A. paniculata* (Burm. f.) Nees (Acanthaceae) (AP) was purchased from a licensed Chinese herbal drug store in Taipei City. The identification of AP was authenticated by Dr.Wei-Chu Li (Sheng Chang Pharmaceutical Co., Ltd) by pharmacognostical anatomical analysis (Chao et al., 2009). The dried whole plant of AP (9 kg) was extracted with 95% ethanol (60 L) at room temperature for 2 weeks. After filtration, 95% ethanol was evaporated under vacuum to obtain a black syrup, which was suspended in water (1 L) and partitioned with EtOAc (1 L three times) to obtain EtOAc-soluble layers (Figure 1).

### 3.2 Fractionation, Isolation, and Identification of Active Compounds From *Andrographis paniculata*

The AP EtOAc-soluble fraction (316.9 g) was separated by silica gel column chromatography eluted by increasing the proportion of EtOAc (0%–100%) in *n*-hexane (Hex) and methanol in EtOAc (10%–50% methanol) to give a total of 26 fractions as described previously (Chao et al., 2009; Chao et al., 2010). The 26 fractions were collected for bioassay-guided fractionation test by measuring their effect on NF-κB-dependent luciferase activity (Chao et al., 2009), and active fractions were collected. After repeated bioassay-guided fractionation by silica gel chromatography, single peak fractions eluted by high-performance liquid chromatography (HPLC) were collected for identification. The chemical compositions of the molecules isolated from AP were analyzed by HPLC and ^13^C NMR and ^1^H NMR spectroscopic data. As shown in Figure 1, compounds **1**, **2**, **3**, **4**, **5**, and **6** were isolated from active fractions (elution with 30%–50% EtOAc/Hex). These were identified as 5-hydroxy-7,8-dimethoxyflavanone (**1**), 5-hydroxy-7,8,2′,5′-tetramethoxyflavone (**2**), cinnamic acid (**3**), ergosterol peroxide (**4**), andrograpanin (**5**), and 14-deoxy-14,15-dehydroandrographolide (**6**). One major component, 14-deoxy-11,12-didehydroandrographolide (**7**), was eluted from 50% EtOAc/Hex.

### 3.3 Synthesis of Analogues of 14-Deoxy-11,12-Didehydroandrographolide (**7**)

**FIGURE 2:**
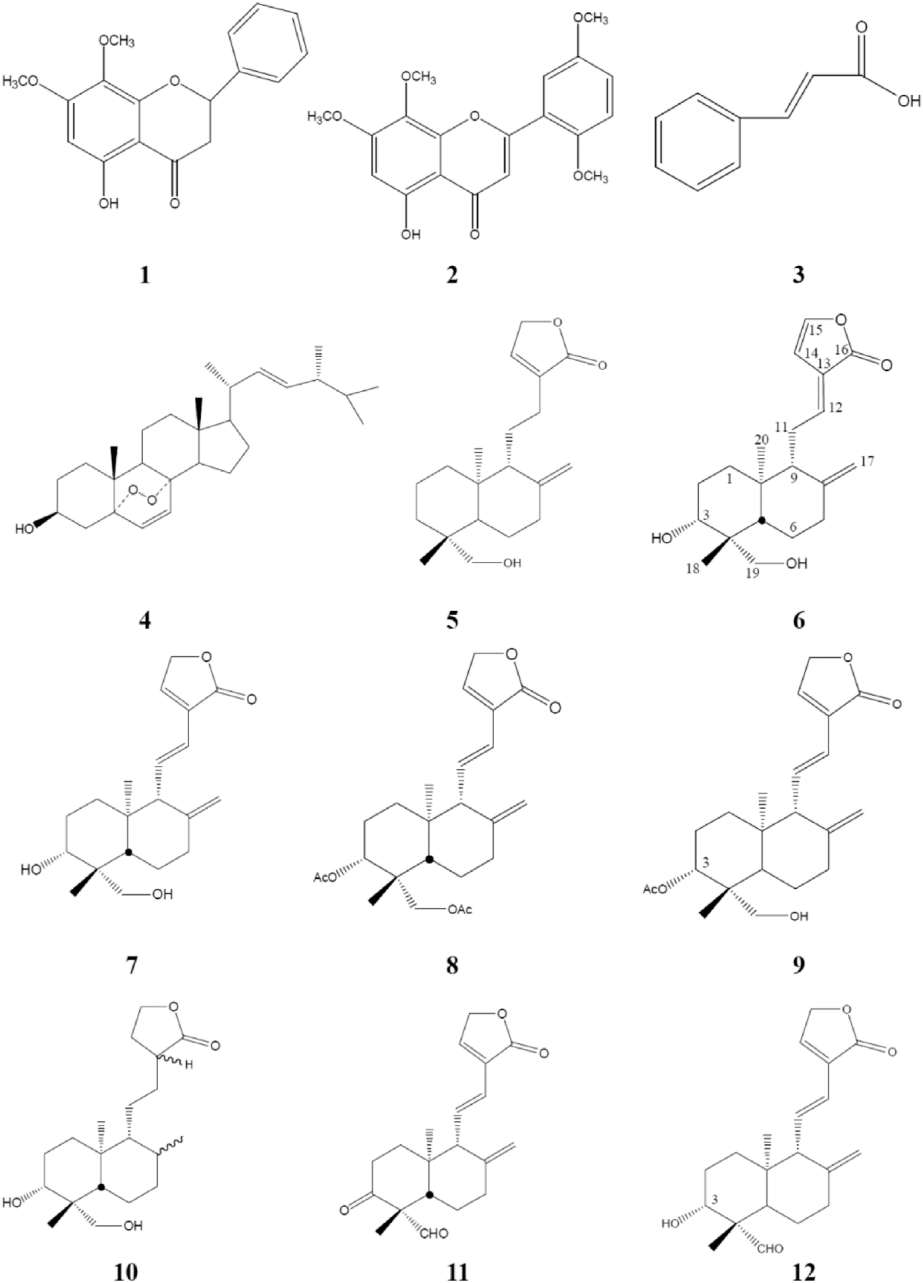
Chemical structures of compounds **1**–**12** isolated or semi-synthesized from AP. AP, *Andrographis paniculata*.

Compound **7** was treated with acetic anhydride in pyridine at room temperature for 1 h (Chao et al., 2010). After the routine workup, compounds **8** and **9** were afforded. For hydrogenation, compound **7** (100 mg) dissolved in 30 ml of acetone with 10% Pd-C (15 mg, as a catalyst) was stirred under a hydrogen atmosphere for 1 h. After filtration and evaporation, the product was purified by HPLC with 50% EtOAc/Hex as the eluted solvent, and compound **10** was yielded. Via Jones oxidation in acetone, compound **7** yielded compounds **11** and **12** (Figure 2). Their chemical structures were elucidated by comparison of their NMR (^1^H and ^13^C) and mass spectrometry as described previously (Chao et al., 2010).

### 3.4 Enterovirus 71 Virus Preparation and Titration

The EV71 virus strain used in this study was TW2272/98 (C2 genogroup, isolated in 1998). Virus strain was propagated in RD cells cultured at 37°C in Alpha Minimum Essential Medium (α-MEM, Invitrogen, Carlsbad, CA, USA) containing 10% fetal bovine serum (FBS). The virus titers were determined based on the CPE developed in infected RD cells and expressed as the median (50%) tissue culture infective dose (TCID_50_) per ml (Lin et al., 2012). Briefly, confluent monolayers of RD cells are plated, and serial dilutions (10^−3^ to 10^−10^) of the virus are added. After incubation for 48 h, the percentage of cell death (i.e., infected cells) is manually observed under microscope, images were recorded as CPE for each virus dilution, and results are used to calculate a TCID_50_ result. The virus was stored at −70°C until use.

The titers of the virus stocks were also determined by a TCID_50_ assay. Serially diluted viruses from 10^−3^ to 10^−10^ in α-MEM with 2% FBS were inoculated to RD cells in 96-well plates, and the cells were incubated for 3 days at 37°C. TCID_50_ was calculated by counting the CPE in infected RD cells. Finally, we selected the infective dose of 10^−7^ EV71 as the median tissue culture infective dose (TCID_50_) in this study to assess the antiviral effect of our samples (Supplementary Figure S1).

### 3.5 Cytopathic Effect Inhibition Assay for Anti-Enterovirus 71-Induced Cytotoxicity of *Andrographis paniculata*

The antiviral activity of AP EtOAc extract against EV71 was determined by a CPE reduction method. RD cells (2 × 10^4^ cell/well) were seeded onto a 96-well culture plate. The next day, the medium was removed, and the cells were pretreated with AP (10–45 μg/ml) for 2 h, then infected with TCID_50_ (10^−7^ dilution) of EV71, and further incubated at 37°C in 5% CO_2_ for 48 h. The morphology of EV71-infected RD cells, with or without pretreatment of AP, was observed under light microscope and recorded as EV71-induced CPE. Infected RD cells in the absence of test compounds were used as the controls. Since test compounds were dissolved in dimethyl sulfoxide (DMSO), 1% DMSO was also added to RD cells for 2 h before infection as solvent control for reference.

### 3.6 Cell Death Analysis by Flow Cytometry

To quantitate death of the infected RD cells, sub-G1 assay by flow cytometry was used to estimate the fractional DNA content. Briefly, a total of RD cells measuring 3 × 10^5^ cells/well were seeded into 6-well culture plates (Falcon; BD Biosciences, San Jose, CA, USA) for 2-h pretreatment with each test compound (2.5–10 μg/ml) and then infected with EV71. The pretreated and infected RD cells were collected 48 h post-infection, washed with phosphate-buffered saline (PBS) buffer, and then centrifuged at 3,000 rpm for 5 min.

The cell pellet was incubated with methanol for 30 min at 4°C, centrifuged again, and washed with PBS buffer. The pellet cells were incubated with RNAase solution, then stained with propidium iodide (PI staining) for DNA content in cell cycle analysis (Sigma Chemical Co., St. Louis, MO, USA), and measured by flow cytometry (FACScan, Becton Dickinson, Mountain View, CA, USA). CellQuest Pro version 4.0 was used for data analysis to calculate the percentage of sub-G1 phase as an indicator of cell death. The increase in sub-G1 cell population in cell cycle indicates cellular apoptosis. The sub-G1 phase increased from 61.6% (10^−7^ EV71) to 86.9% (10^−6^ EV71) when more EV71 was added, consistent with microscopic images of CPE (Supplementary Figure S1).

### 3.7 IFNγ-Luciferase Reporter Gene Assay

To investigate whether these compounds possess IFN-inducing activity, the transient transfection assay using an IFNγ-luciferase reporter gene was performed. EL-4 T cells, a murine T lymphocyte cell line, grown in Dulbecco’s modified Eagle’s medium (DMEM) with 10% FBS were seeded on 24-well plates at a concentration of 4 × 10^5^ cells/well. The EL-4 T cells were transiently transfected with 0.9 μg of pIFNγ-luc, a plasmid containing IFNγ promoter with luciferase reporter gene, and 0.1 μg of internal control pRL-tk plasmid for 5 h, as described previously (Chao et al., 2009). EL-4 transfectants were pretreated with each test compound (2.5–10 μg/ml) or vehicle for 1 h and then stimulated without or with phorbol 12-myristate 13-acetate (PMA; 50 ng/ml, Sigma)/ionomycin (1,000 ng/ml, Sigma) for 24 h. Cell lysis was performed, and luciferase activity measured as previously reported (Chao et al., 2009).

### 3.8 Statistical Analysis

The data were expressed as mean ± SD. The significant difference compared with the control group was analyzed by Student’s t-test using the SAS statistical software (SAS/STATA version 8.2; SAS Institute, Cary, NC, USA). The difference was considered to be significant if *p* was <0.05.

## 4 Results

### 4.1 Structures of Pure Compounds **1**–**12** **From** ***Andrographis paniculata*** **Ethyl Acetate Extract**

Isolation and identification of active compounds from AP are as shown in Figure 1. The chemical structures of compounds **1**–**12** isolated or semi-synthesized from AP EtOAc extract are illustrated in Figure 2. The compounds selected for this study are as follows: flavonoids 5-hydroxy-7,8-dimethoxyflavanone (**1**) (29 mg) and 5-hydroxy-7,8,2′,5′-tetramethoxyflavone (**2**) (169.7 mg); acid cinnamic acid (**3**) (63.3 mg); steroid ergosterol peroxide (**4**) (10.2 g); diterpenoids andrograpanin (**5**) (676.3 mg) and 14-deoxy-14,15-dehydroandrographolide (**6**) (1.2 g); and one major bioactive component 14-deoxy-11,12-didehydroandrographolide (**7**) (14.7 g).

Five synthetic analogues from this major compound **7** are as follows: 3,19-*O*-acetyl-14-deoxy-11,12-didehydroandrographolide (**8**) (72.6 mg) and new compound 3-*O*-acetyl-14-deoxy-11,12-didehydroandrographolide (**9**) (3.3 mg) by acetylation; new compound hexahydro-14-dehydroxyandrographolide (**10**) (22.4 mg) by hydrogenation; and new compounds 3,19-dioxolabda-8(17),11*E*,13-trien-16,15-olide (**11**) (63.2 mg) and 3α-hydroxy-19-oxolabda-8(17),11*E*,13-trien-16,15-olide (**12**) (5.4 mg) by oxidation.

The major compound 14-deoxy-11,12-didehydroandrographolide (**7**) is known to possess immunostimulatory and anti-atherosclerotic activities and is anti-inflammatory anti-infective (Cai et al., 2020). Andrograpanin is a minor compound but was also reported to have both anti-inflammatory and anti-infective properties (Chao and Lin, 2010; Chandrasekaran et al., 2011). In a previous study, Chao et al. showed that AP presented as an inflammatory inhibitor through the suppression of NF-κB signaling (Chao et al., 2010; Tung et al., 2010). Therefore, the antiviral activity of the major compounds is worthy of further investigation.

### 4.2 Protection of Enterovirus 71-Induced Cytopathic Effect With *Andrographis paniculata* Extract

**FIGURE 3:**
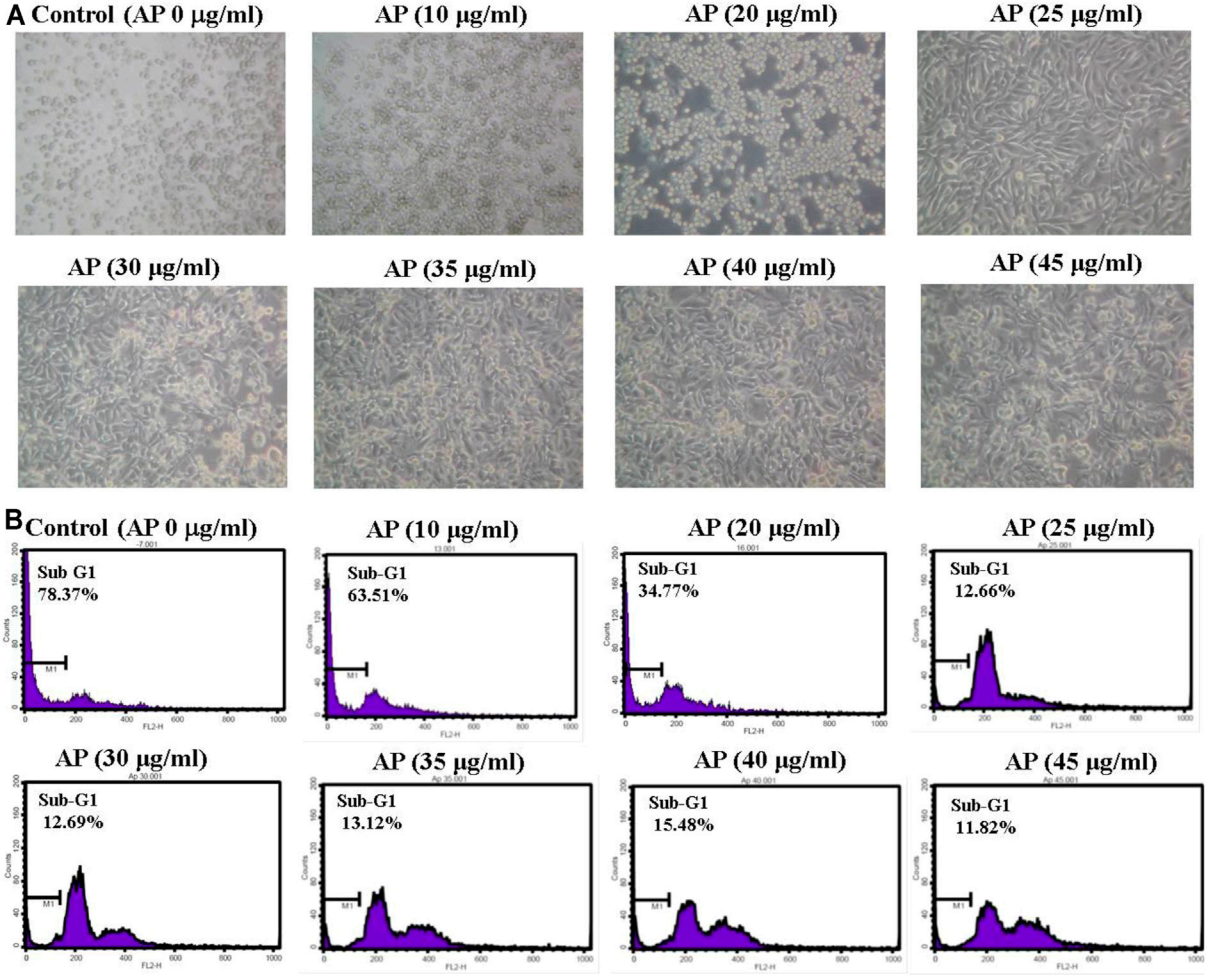
EV71-induced cytopathic effects are inhibited by AP EtOAc extracts. **(A)** Morphological changes of RD cells observed under an inverted microscopy (×20). **(B)** Sub-G1 population of the infected RD cells was analyzed by flow cytometry illustrated as a histogram. Confluent monolayers of RD cells were pretreated with AP EtOAc extracts at the doses of 10, 20, 25, 30, 35, 40, or 45 μg/ml for 2 h and then challenged with 10^−7^ of EV71 simultaneously at 37°C. The cytopathic effect was observed under a microscope (×20) after 48 h. Then, the cells were trypsinized, and sub-G1 cell cycle arrest was analyzed in flow cytometry after being stained with PI. M1 indicates the sub-G1-gated region in the histogram. Data are representative of three independent experiments. All AP-treated results were significantly different from those of EV71 only analyzed by Student’s *t*-test (Supplementary Table S1). EV71, enterovirus 71; AP, *Andrographis paniculata*; EtOAc, ethyl acetate; RD, rhabdomyosarcoma; PI, propidium iodide.

The antiviral activities of AP EtOAc extract against EV71 are based on inhibition of virus-induced CPEs in RD cells. AP EtOAc extract was first confirmed to have no cytotoxicity against host cells at concentrations up to 45 μg/ml. RD cells were pretreated with a variety of concentrations of AP EtOAc extract for 2 h before EV71 inoculation. As shown in Figure 3, EV71 infection increased the apoptotic rate of RD cells as indicated in 78.4% sub-G1 phase. When treated with different concentrations higher than 10 μg/ml of AP, the apoptotic rates of RD cells decreased from 63.5% to 11.8% (Figure 3). The antiviral assays demonstrated that AP EtOAc extract could significantly inhibit the CPE of EV71 viral infection.

### 4.3 Inhibition of Enterovirus 71-Induced Cytopathic Effect by Compounds **1**–**12** **From** ***Andrographis paniculata***

To further investigate bioactive compounds fractionated and purified from AP that exert anti-EV71 effects, compounds **1**∼**12** obtained by bioassay-guided fractionation of a 95% ethanol extract of AP, as shown in Figures 1 and 2, were tested for anti-EV71 activity. Compounds **1**∼**12** dissolved in DMSO at the concentrations of 2.5, 5, and 10 μg/ml without any cytotoxic effect were used for the anti-EV71-induced cytotoxicity test. The results are summarized in Table 1, as categorized into the following subgroups: flavones, acid, steroids, terpenoids, and synthetic analogues of compound **7**.

**TABLE 1:** Anti-EV71-induced cytotoxicity of AP EtOAc extract and its pure compounds evaluated by the decrease in percentage of sub-G1 population in EV71-infected RD cells.

	Sub-GI (%)		
**EV71 only**	64.96 ± 4.82		
**2% FBS–alpha-MEM medium only**	4.46 ± 1.63*		
**1% DMSO only**	4.05 ± 1.10*		
**EV71 with extract or pure compounds**	2.5 μg/ml	5 μg/ml	10 μg/ml
AP EtOAc extract Flavones	nd	nd	57.21 ± 8.92
5-Hydroxy-7,8-dimethoxyflavanone **(1)**	39.96 ± 0.35*	20.54 ± 0.66*	7.60 ± 0.57*
5-Hydroxy-7,8,2′,5′-tetramethoxyflavone **(2)**	37.14 ± 3.34*	19.12 ± 1.25*	9.11 ± 0.64*
Phenolic acid			
Cinnamic acid **(3)**	25.41 ± 1.97*	14.39 ± 0.86*	8.55 ± 0.92*
Steroids			
Ergosterol peroxide **(4)**	14.99 ± 2.14*	10.47 ± 0.75	8.15 ± 2.45*
Diterpenoids			
Andrograpanin **(5)**	21.25 ± 1.91*	13.71 ± 1.82*	8.33 ± 1.56*
14-Deoxy-14,15-dehydroandrographolide **(6)**	33.24 ± 1.18*	26.40 ± 4.31*	10.85 ± 1.63*
14-Deoxy-11,12-didehydroandrographolide **(7)**	26.44 ± 4.61	21.89 ± 0.87*	15.29 ± 3.20*
Synthetic analogues			
3,19-*O*-Acetyl 11,12-didehydroandrographolide **(8)**	18.24 ± 2.77	9.16 ± 2.32*	5.61 ± 1.70*
3-*O*-Acetyl 14-didehydroandrographolide **(9)**	63.16 ± 0.50*	37.15 ± 1.62*	17.14 ± 3.42*
Hexahydro-14-dehydroxyandrographolide **(10)**	60.23 ± 5.61*	40.58 ± 0.82*	28.08 ± 1.27*
3,19-Dioxolabda-8(17),11*E*,13-trien-16,15-olide **(11)**	57.34 ± 2.74*	34.03 ± 1.45*	22.99 ± 1.43*
3α-Hydroxy-19-oxolabda-8(17),11E,13-train-16,15-olide **(12)**	36.55 ± 3.05*	21.86 ± 1.50*	18.87 ± 0.33*

The sub-G1-gated region by flow cytometry indicates cells undergoing apoptotic changes. Values are expressed as means ± SD of three independent experiments with three replicates in each experiment and statistically analyzed by Student’s t-test.

*p < 0.05 indicates a significant difference from EV71 only.

nd, not determined; EV71, enterovirus 71; AP, Andrographis paniculata; EtOAc, ethyl acetate; RD, rhabdomyosarcoma; FBS, fetal bovine serum; DMSO, dimethyl sulfoxide.

As the antiviral activity is evaluated by the decrease in percentage of sub-G1 phase, the results repeated the protective effects of 10 μg/ml of AP extract in EV71-infected RD cells, as shown in Figure 3, with 63.51% of apoptotic populations. The isolated compounds **1**∼**7** decreased sub-G1 percentages to exert 38%–77% protection of EV71-induced CPEs compared with EV71 only control at concentration of 2.5 μg/ml (Table 1). Ergosterol peroxide (**4**) showed the strongest protection among compounds **1**∼**7**, with 77%–88% inhibitory effect of apoptosis at the concentration of 2.5–10 μg/ml addition to EV71-infected RD cells. At a higher concentration of 10 μg/ml, most of these compounds had 88% inhibition except for compounds **6** and **7**, which exerted 76%–83% inhibition of apoptosis caused by EV71.

Since compound **7** was the major compound isolated from AP EtOAc extract, further modifications were attempted to improve its antiviral effect. The synthetic analogues shown in Table 1 demonstrated that the EV71-induced sub-G1 phase was reduced from 65.0% to 5.6% when compound **8** at the concentration of 2.5–10 μg/ml was added to the infected cells. 3,19-*O*-Acetyl-14-deoxy-11,12-didehydroandrographolide (**8**) had much lower sub-G1 population than 14-deoxy-11,12-didehydroandrographolide (**7**), indicating its stronger protection from EV71 infection through chemical modification of compound **7**.

### 4.4 Effects of Compounds **1**∼**12 on Activation of IFNγ in EL-4 T-Cell Line**

**FIGURE 4:**
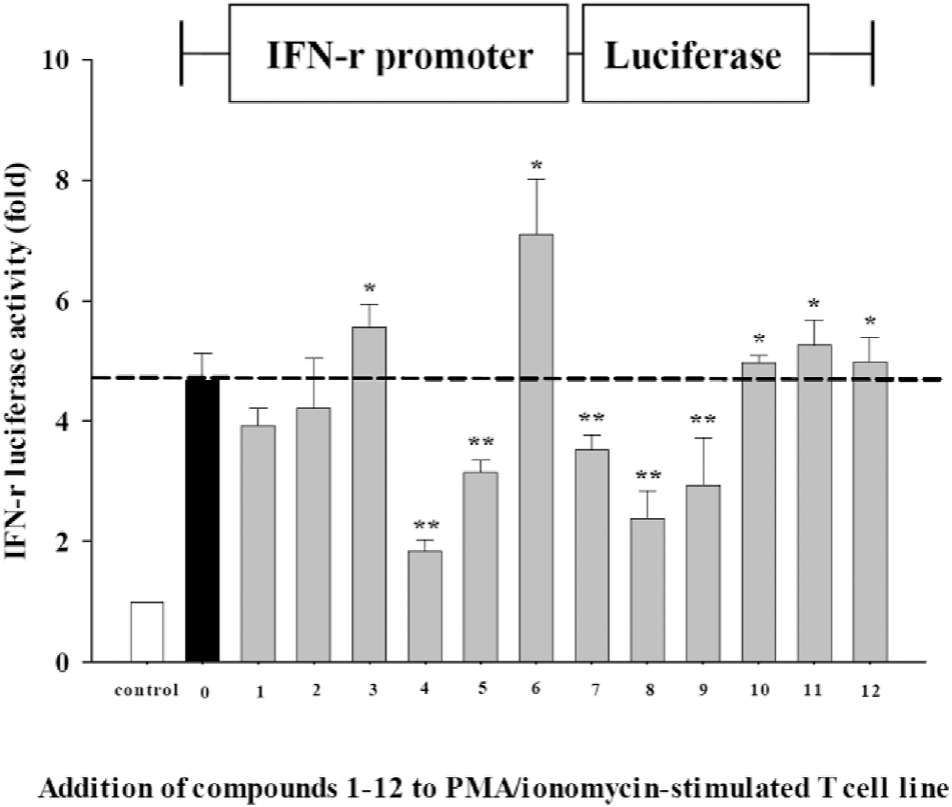
IFNγ-luciferase activity compounds **1**∼**12** from AP EtOAc extract. EL-4 T cells transfected with IFNγ-luciferase reporter gene were stimulated with PMA (50 ng/ml)/ionomycin (1,000 ng/ml) in the absence or presence of 12 test compounds. Data representative of three independent experiments are expressed as mean ± SD. IFNγ-luciferase activity is represented as a fold ratio to the control without stimulation (white bar). *p < 0.05; **, *p* < 0.01 vs. stimulated cells in the absence of test compound (black bar). AP, *Andrographis paniculata*; EtOAc, ethyl acetate; PMA, phorbol 12-myristate 13-acetate.

Since IFNs are important not only to combat virus infection but also to modulate the antiviral immune responses, we further employed an IFNγ promoter-driven luciferase reporter construct to investigate whether these pure compounds also exert IFNγ-driven activity, using luciferase reporter gene expression. As shown in Figure 4, as compared with those cells incubated with medium without stimulation (white bar), incubation of EL-4 T cells with PMA/ionomycin increased IFNγ transactivation activity (black bar, *p* < 0.05). The ratio of luciferase intensity revealed that cinnamic acid (**3**) and 14-deoxy-14,15-dehydroandrographolide (**6**) might have IFNγ induction potential to activate its promoter. In addition, three of chemically modified analogues of compound **7**, hexahydro-14-dehydroxyandrographolide (**10**), 3,19-dioxolabda-8(17),11*E*,13-trien-16,15-olide (**11**), and 3α-hydroxy-19-oxolabda-8(17),11*E*,13-trien-16,15-olide (**12**), also significantly increased the promoter activity. It suggests a potential action of these compounds to induce IFNγ transcription. In contrast, compounds **4**, **5**, **7**, **8**, and **9** showed inhibitory effects on IFNγ transcription.

## 5 Discussion

EV71 is a pathogen causing many disease symptoms in humans, especially infants and children under the age of 5. Unfortunately, there is no effective vaccine for prevention or antiviral drug against EV71 infection (Tosh et al., 2020). Thus, there is a need to develop effective antiviral agents to treat EV71 infection.

With a combined effort of bioassay-guided purification, high-resolution mass spectrometry, and NMR, we identified the fractions of AP responsible for anti-EV71-induced cytotoxicity *in vitro*. For the first time, the pure compounds isolated from AP, 5-hydroxy-7,8-dimethoxyflavanone (**1**), 5-hydroxy-7,8,2′,5′-tetramethoxyflavone (**2**), cinnamic acid (**3**), ergosterol peroxide (**4**), andrograpanin (**5**), 14-deoxy-14,15-dehydroandrographolide (**6**), and 14-deoxy-11,12-didehydroandrographolide (**7**), were found to inhibit EV71-induced CPE, which represent anti-EV71 infection activities. In addition, after acetylation, hydrogenation, and oxidation of compound **7**, compound **8** was found to have the strongest antiviral activity (Table 1), suggesting that chemical modification of major compounds for improvement of potency is worthy of further investigation.

In addition to the evaluation of anti-EV71-induced cytotoxicity by inhibiting apoptosis of EV71-infected RD cells, the effects on IFNγ-activation were also investigated. IFNγ plays a vital role in stimulating immune response, primarily secreted by activated T cells and natural killer cells. We investigated the IFNγ-inducing effects of these 12 pure compounds by using a T lymphocyte cell line transfected with IFNγ reporter gene. Among these compounds, we identified cinnamic acid (**3**), 14-deoxy-14,15-dehydroandrographolide (**6**), and synthetic analogues hexahydro-14-dehydroxyandrographolide (**10**), 3,19-dioxolabda-8(17)(11)*E*,13-trien-16,15-olide (**11**), and 3α-hydroxy-19-oxolabda-8(17),11*E*,13-trien-16,15-olide (**12**) with IFNγ-inducing activity, which is crucial for viral clearance in virus-infected tissues (Stubblefield Park et al., 2011). Cinnamic acid is an organic chemical mainly isolated from cinnamon. Natural and synthetic cinnamic acid derivatives were reported to exhibit multiple biological activities including anti-inflammatory, antimicrobial, anti-oncogenic, antioxidant, kinase-inhibitory effects, and/or inhibit hepatitis C virus replication (Mielecji and Lesyng, 2016; Amano et al., 2017).

IFNγ not only induces antiviral immune response but also activates macrophage to increase phagocytosis and production of inflammatory mediators (Lee and Ashkar, 2018). Therefore, compounds **4**, **5**, **7**, **8**, and **9** with decreased IFNγ transcription imply that inhibition of IFNγ expression might be beneficial for anti-inflammation at the immune homeostatic phase of the battle of host *vs.* virus. This dual nature of IFNγ not only exerts antiviral immune response by compounds **3** and **6** to limit virus replication but also negatively regulates this response by compounds **4**, **5**, **7**, and **8** to avoid further tissue damage. Ergosterol peroxide can inhibit porcine deltacoronavirus (PDCoV) infection and regulate host immune responses (Hong et al., 2009; Duan et al., 2021). The enhancement of peripheral blood lymphocyte proliferation and IL-2 secretion that activates immune cells by 14-deoxy-11,12-didehydroandrographolide (**7**) might also contribute to the antiviral function (Kumar et al., 2004). Zhou et al. (2019) showed that ergosterol peroxide has been shown to exhibit antitumor, antioxidant, anti-bacterial, and anti-influenza A virus properties (Zhou et al., 2019).

TCM has its perspective and unique advantages derived from its 2,500-year history. Since the 1950s, the chemical components of *A. paniculata* have been well investigated. *A. paniculata* is a heat-clearing and detoxifying medicine. According to the present investigation, diterpenoid lactones (34.95%) and flavonoids (46.23%) are the major classes of chemical compounds especially from aerial parts (61.93%) of *A. paniculata*. Other classes, such as terpenoids (10.22%), phenolic acids (4.30%), chalconoids (2.15%), xanthones (2.15%), and volatile compounds, are also reported in different plant parts (Chao and Lin, 2010; Wang et al., 2016; Jiang et al., 2021; Kumar et al., 2021).

Natural products have played pivotal roles in the drug discovery and development process. For antivirus effects of AP, its ethanol extract was reported to alleviate inflammation in H1N1-infected human bronchial epithelial cells by inhibiting chemoattractant activity (Ko et al., 2006). Andrographolide is a major bioactive component of the plant; a labdane diterpenoid has been reported for anti-hepatitis virus activity (Chen et al., 2014; Sa-hgiamsuntorn et al., 2021). In addition, through chemical modification, the synthesized derivatives of andrographolide could enhance its anti-HIV effect (Reddy et al., 2005). 14-Deoxy-11,12-didehydroandrographolide (**7**) was also demonstrated to exert anti-HIV activity *in vitro* (Uttekar et al., 2012), attenuated excessive inflammatory response, and protected mice lethally infected with H5N1 virus *in vivo* (Cai et al., 2016). We further chemically modified compound **7** to synthesize 3,19-*O*-acetyl-14-deoxy-11,12-didehydroandrographolide (**8**) and found stronger anti-EV71-induced cytotoxicity than that of compound **7**. It is suggested that the application of plants could be diversified into the major compound for its therapeutic targets and the other less abundant compounds for modification to increase their efficacy. 14-Deoxy-11,12-dehydroandrographolide is one of the major active constituents of *A. paniculata*. Studies found that 14-deoxy-11,12-dehydroandrographolide strongly inhibited H5N1 replication (Cai et al., 2016; Cai et al., 2020).

In our study, based on the information of structure–activity relationships (Chen et al., 2014; Nguyen et al., 2015), the acetylation, hydrogenation, and oxidation were performed to modify the most abundant compound isolated. We have successfully modified the hydroxyl groups at C-3 and C-19 of compound 14-deoxy-11,12-didehydroandrographolide (**7**). Compounds **8** and **9** were obtained from acetylation of 14-deoxy-11,12-didehydroandrographolide (**7**), and thus, 3,19-*O*-acetyl- in (**8**) exerted more inhibitory effects than 3-*O*-acetyl- in (**9**), which indicated that 19-acetyl did make a difference. Compound **10** obtained from hydrogenation of (**7**) have hexahydro- that saturated three double bonds. Compound **10** exerting less effect indicated that the three double-bond structures were critical. Compounds **11** and **12** obtained from oxidation of (**7**) both have 19-oxo-, but compound **11** oxidized to 3-keto showed less effect than compound **12**, indicating its effective structure order 3-acetyl- > 3-hydroxy- > 3-keto.

Several natural products, herbs, or synthetic compounds have been found to display antivirus infection (Ho et al., 2009; Santangelo et al., 2012; Zhang et al., 2014). For example, the viral CPE on RD cells can be reduced by inhibiting virus replication and further confirmed by the low mortality of mice challenged with a lethal dose of EV71, by several compounds such punicalagin (Yang et al., 2012), a component of pomegranate (*Punica granatum* L.). Chrysosplenetin and penduletin, *O*-methylated flavonols isolated from the leaves of *Laggera pterodonta*, were demonstrated to block virus entry or replication in RD cells (Zhu et al., 2011). The inhibition of EV71 VP1 protein production by several compounds such as glycyrrhizic acid isolated from *Glycyrrhiza uralensis* (Wang et al., 2013), formononetin (a kind of plant isoflavonoid from red clover) (Wang et al., 2016), and luteoloside, curcumin, and quercetin from a number of plants and herbs (Cao et al., 2016; Huang et al., 2018; Yao et al., 2018). All these studies indicated great potential of compounds isolated, and further synthesis of analogues from plants has anti-EV71-induced cytotoxicity.

In our study, we evaluated anti-EV71-induced cytotoxicity of herbal compounds by measuring sub-G1 percentage of infected RD cells as the apoptotic response caused by virus. We first studied the anti-EV71 activity of AP EtOAc extracts, and we further investigated seven pure compounds isolated from this extract and five synthetic analogues of the compound in significant amount. Ergosterol peroxide (**4**) had the highest anti-EV71 activity. Further, 3,19-*O*-acetyl-14-deoxy-11,12-didehydroandrographolide (**8**) showed increased anti-EV71 activity after chemical modification of one major bioactive component 14-deoxy-11,12-didehydroandrographolide (**7**). By transfection of IFNγ report gene to T-cell line for transactivation assay, cinnamic acid (**3**) and 14-deoxy-14,15-dehydroandrographolide (**6**) were found to have IFNγ-inducing effect; and ergosterol peroxide (**4**), andrograpanin (**5**), 14-deoxy-11,12-didehydroandrographolide (**7**), 3,19-*O*-acetyl-14-deoxy-11,12-didehydroandrographolide (**8**), and 3-*O*-acetyl-14-deoxy-11,12-didehydroandrographolide (**9**) could suppress IFNγ expression in T cells, which might be critical for anti-inflammatory activity of these compounds. Furthermore, chemical modification of known active natural compounds may lead to better structural optimization to yield higher efficiency and lower toxicity, thus promoting anti-EV71 drug development. Our study demonstrated that bioactive compounds of AP and its derivatives either protecting EV71-infected RD cells from sub-G1 arrest or possessing IFNγ-inducer activity might be feasible for the development of anti-EV71 agents.

**FIGURE 5:**
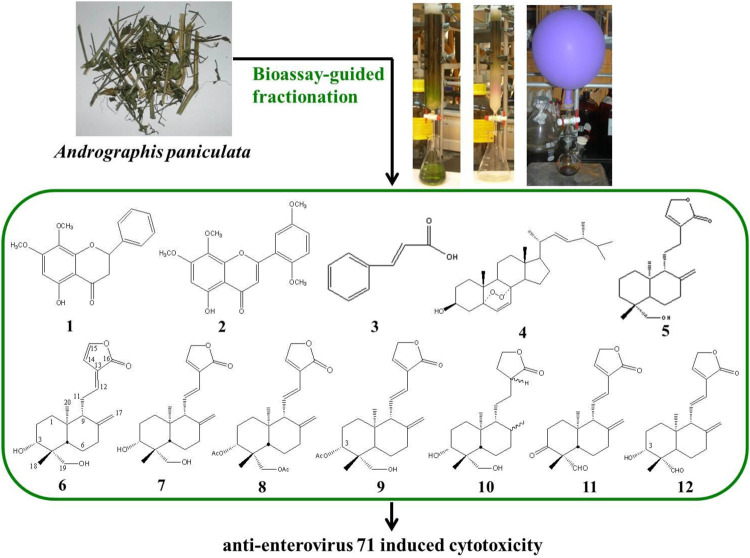
Schematic summary of the current study.

In summary, this plant is reported to have diterpenoids, flavonoids, and steroids. One or more active ingredients can act alone or in synergy in this extract (Figure 5). The results in this study demonstrated the anti-EV71-induced cytotoxicity of compounds isolated from *A. paniculata*, and further chemical modification of the compounds could increase the antiviral activity or IFNγ-inducing activity.

## Data Availability

The original contributions presented in the study are included in the article/Supplementary Material, Further inquiries can be directed to the corresponding authors.
