# Supplementary material for: Isolation and Identification of Andrographis paniculata (Chuanxinlian) and Its Biologically Active Constituents Inhibited Enterovirus 71-Induced Cell Apoptosis
Source: Front Pharmacol. 2021 Dec 8;12:762285. doi: 10.3389/fphar.2021.762285 (PMC8692857; doi:10.3389/fphar.2021.762285)
Supplement: Supplementary file 1 [file DataSheet1.docx]

Supplementary Material

# Supplementary Data

**Determination of EV71-Induced Cytopathic Effect (CPE)**

RD cell line is widely accepted model for the assay of enteroviruses because it is sensitive to EV71 infection. First, the microscopic examination of unstained cell culture was checked since it has long been the standard approach for detecting viral infection. As shown in **Supplementary Figure A**, RD cells were approximately 80% confluence after 2 days of incubation without or with DMSO, the solvent for test compounds in this study. When EV71 was added, EV71-infected RD cells rounded up and exhibited a typical tear-like CPE, as shown in 10^-8^, 10^-7^, or 10^-8^ EV71-infected RD cells.

Apoptosis is a common cellular response to viral infection and a major pathogenic mechanism of viruses, including EV71. Recent study demonstrated that EV71-induced cell death was mainly executed by apoptosis (Bai et al., 2019). In RD cells, apoptosis was clearly observed upon infection with EV71 and quantitated by flow cytometer, as sub-G1 peak cells. Our results show that sub-G1 phase increased from 3.3% (RD cell only or treated with DMSO) to 22.6% by addition of 10^-8^ EV71 (**Supplementary Figure B**). The sub-G1 phase increased to 61.6% (10^-7^ EV71) and 86.9% (10^-6^ EV71) when more EV71 were added, consistent with microscopic images of CPE. Finally, we selected the infective dose of 10^-7^ EV71 as the median tissue culture infective dose (TCID_50_) in this study to assay antiviral effect of our samples.

# Supplementary Figures and Tables

## Supplementary Figures

**
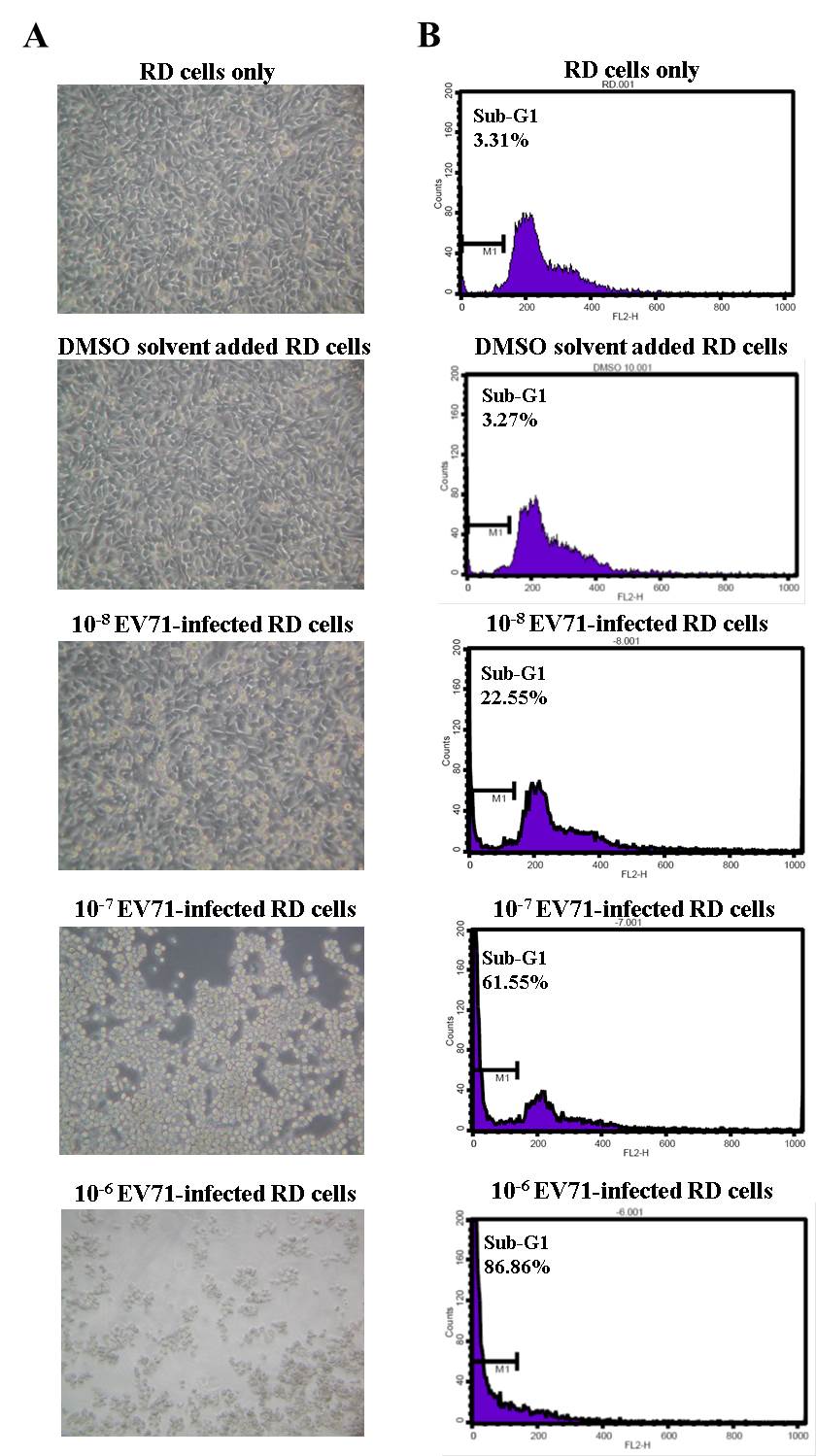
**

**Supplementary Figure 1.** The cytopathic effect on RD cells and its apoptosis analysis with or without inoculation of EV71. Different diluted virus suspensions 10^-6^, 10^-7^, 10^-8^ of EV71 was added. **(A)**Morphological changes of RD cells observed under an inverted microscopy (x20). **(B)**Sub-G1 population of the infected RD cells were analyzed by flow cytometry illustrated as histogram. The FL-2 represents the intensity of PI staining analysis. The peak representing the sub-G1 phase is characteristic of cells undergoing apoptosis. M1 indicated the sub-G1 gated region.

**Supplementary Table** Ssub-G1 percentage of EV71-induced cytopathic effects inhibited by AP EtOAc extracts


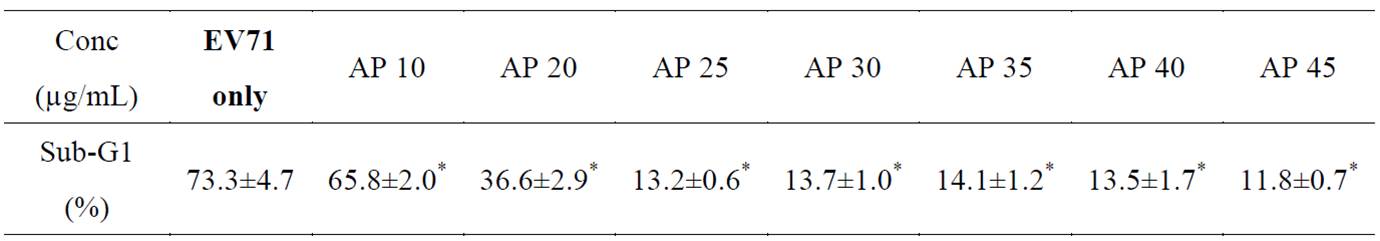


Data are mean ± SD (n=3). All AP-treated results were significantly different from EV71 only analyzed by Student’s *t*-test.
